# Germinated *Rhynchosia nulubilis* Fermented with *Lactobacillus pentosus* SC65 Reduces Particulate Matter Induced Type II Alveolar Epithelial Apoptotic Cell Death

**DOI:** 10.3390/ijms22073660

**Published:** 2021-04-01

**Authors:** Hye-Ji Lee, Hye-Jin Park

**Affiliations:** Department of Food Science and Biotechnology, College of BioNano, Gachon University, 1342 Seongnam-daero, Sujeong-gu, Seongnam-si 461-701, Gyeonggi-do, Korea; manda1002@naver.com

**Keywords:** PM < 11, ROS, A549 cells, cell death, apoptosis, germinated *Rhynchosia nulubilis* fermented with *Lactobacillus pentosus* SC65

## Abstract

Particulate matter (PM) is a significant environmental pollutant that promotes respiratory diseases, including lung injury and inflammation, by inducing oxidative stress. *Rhynchosia nulubilis* (black soybean) is traditionally used to prevent chronic respiratory disease via inducing antioxidant and anti-inflammatory effects. To investigate the effects of *Lactobacillus pentosus* SC65 fermented GR (GR-SC65) and *Pediococcus pentosaceus* ON81A (GR-ON81A) against PM-induced oxidative stress and cell death in A549 cells, we performed the 2-7-dichlorodihydrofluorescein diacetate and cell counting kit-8 assays, as well as Hoechst 33342 and propidium iodide staining and western blotting. GR-SC65 showed the highest total polyphenolic contents and 1,1-diphenyl-2-picrylidrazil radical scavenging activity among lactic acid bacteria-fermented GRs (*p* < 0.001 vs. GR). Four soy peptides, β-conglycinin breakdowns (INAENNQRNF, ISSEDKPFN, LAFPGSAQAVEK, and LAFPGSAKDIEN), were detected in GR-SC65, but not in GR. In GR-SC65, PM-induced A549 cell death was less than that observed in GR-ON81A and GR (*p* < 0.001 vs. PM-treated group). GR-SC65 significantly decreased intracellular reactive oxidative species (ROS) when compared with PM (*** *p* < 0.001 vs. PM). GR-SC65 decreased the levels of BAX, active caspase-9, -3, and poly ADP-ribose polymerase (PARP) proteins (^#^ *p* < 0.01, ^###^ *p* < 0.001 vs. PM), while increasing the level of BCL-2 protein, a mitochondrial anti-apoptotic protein (^###^ *p* < 0.001 vs. PM). Our findings indicate that GR-SC65 inhibited PM-induced cell death by suppressing the levels of ROS, active caspase-9 and -3, and PARP proteins, while enhancing the level of BCL-2 protein in type II alveolar epithelial A549 cells. Therefore, GR-SC65 might be a potential therapeutic and preventive agent against PM-induced lung injury.

## 1. Introduction

Reportedly, ambient airborne particulate matter (PM) has rapidly increased the rate of respiratory, skin, and cardiopulmonary diseases in China, Korea, and Japan [1]. Over the last few decades, epidemiological studies have consistently illustrated that there exists a strong correlation between the levels of fine particles (diameter < 2.5 μm (PM2.5) and diameter < 10 μm (PM10)) and mortality rates, owing to respiratory disease. In recent years, the hazardous effects of PM2.5 have increasingly drawn public concern [2]. PM2.5 can penetrate deeper into the airways of the respiratory tract and alveoli, increasing the incidence of myocardial infarction and lung cancer in adults, as well as asthma in children and adults [3,4]. Notably, oxidative stress is the main mechanism underlying PM-related diverse pathological events [4]. PM can induce either the excessive generation of reactive oxygen species (ROS) or impair endogenous antioxidants, resulting in lung inflammation and injury of the respiratory tract and other lung tissues [5]. It is crucial that novel methods are developed to afford protection against the harmful health-related effects of PM2.5. Food materials possessing antioxidant properties have gained momentum as potential therapeutic agents against PM2.5-induced diseases.

Herbal medicines or natural foods to prevent lung damage caused by PM have attracted considerable public attention. For instance, red ginseng, algae, and water celery (*Oenanthe javanica*) are known therapeutic or preventive foods for lung damage [6,7]. However, clinical studies are needed to determine the effects of therapeutic or preventive foods on lung damage.

Soybean (*Glycine max*) and its products, including fermented soybean sauces, are widely consumed in East Asia. For instance, *Rhynchosia nulubilis* (black soybean; RN) is traditionally used to prevent chronic respiratory diseases owing to its antioxidant and anti-inflammatory effects [8,9,10]. Several bioactive constituents such as proteins, soyasaponins, isoflavones, pro-anthocyanidins, and polysaccharides are found in soybeans, which are known to possess health-promoting effects [11,12]. Soybean phenolic compounds, including isoflavones and pro-anthocyanidins, reportedly prevent chronic diseases owing to their antioxidant and anti-inflammatory effects [13,14,15,16]. RN, the so-called Yak-Kong, is a soybean cultivar with a black seed coat and green embryo. It is used as a medicinal herb owing to its antioxidant and anti-inflammatory effects [12]. Takahashi et al. have observed that RN shows stronger antioxidant activity than common black and yellow soybeans. Isoflavones (genistein, daidzein, and glycitein) in soybeans are known to decrease ROS levels by scavenging and suppressing their intracellular production [17]. Phenolic compounds are known to enhance the antioxidant capacity of cells by stimulating superoxide dismutase (SOD), catalase (CAT), glutathione peroxidase (GPx), and xenobiotic detoxification enzymes [2].

Fermentation is a traditional method for the production of functional foods that increase the production of valuable bioactive compounds. Lactic acid bacteria (LAB) have beneficial effects such as antioxidant, anti-inflammatory, and anti-allergic activities by increasing the formation of phenolic acids and aglycone forms of isoflavones [18,19]; however, reports on germinated RN fermented with LAB for the prevention of PM-induced lung diseases are lacking. Previously, we sought to determine the optimal LAB for RN fermentation [20]. As it is well-known that PM-induced oxidative stress can induce various lung diseases, we hypothesized that if RN metabolites produced by LAB fermentation increase the antioxidant activity, this would prevent PM-induced lung diseases by regulating oxidative stress and related enzymes/signaling molecules. In the present study, we investigated whether LAB-fermented germinated RN extract could suppress PM-induced cell death and oxidative stress in the type II alveolar epithelial cell line, A549.

## 2. Results

### 2.1. LAB Fermentation Increases Total Polyphenolic Content and DPPH Radical Scavenging Activity in GR

To select the best LAB strain for fermenting GR, TPC and DPPH radical scavenging activities were compared with those of GR-SC65 and GR-ON81A. Polyphenol compounds are known to exert antioxidant activity [21,22,23,24]. To determine the extraction temperature, the TPC in GR, GR-SC65, and GR-ON81A were compared after extraction at 27 °C and 50 °C (Table 1). In each sample, the TPC was higher following extraction at 50 °C (GR 80.9 ± 0.6; GR-SC65, 113.2 ± 2.3; GR-ON81A, 89.8 ± 0.2) than at 27 °C (GR, 93.8 ± 1.3; GR-SC65, 161.6 ± 1.9; GR-ON81A, 158.8 ± 2.8). Reportedly, higher extraction temperatures enhance the solubility of polyphenols owing to a decrease in solvent viscosity and an increase in molecular movement [25].

**Figure 1:**
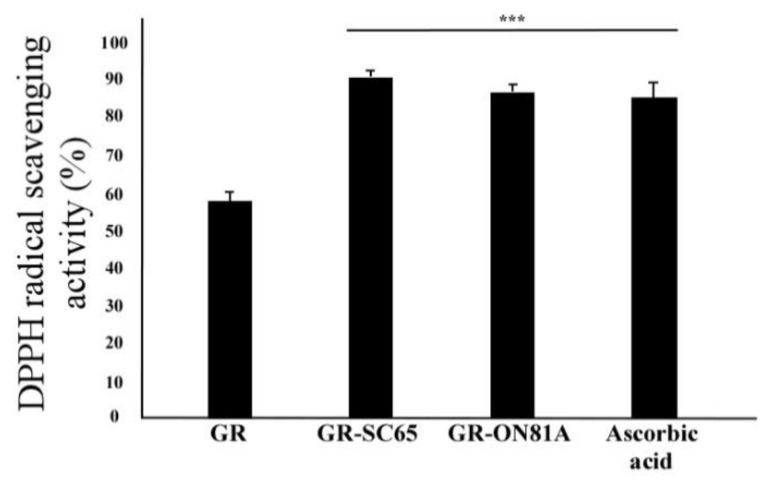
Comparison of diphenyl picrylhydrazyl (DPPH) radical scavenging activity in GR fermented with different lactic acid bacteria strains (*L. pentosus* SC65 and *P. pentosaceus* ON81A). Results from three independent experiments are expressed as means ± standard deviation (SD). Statistically significant differences between groups were analyzed using one-way ANOVA/Dunnett’s *t*-test (*** *p* < 0.001 vs. GR).

The antioxidant activities of GR, GR-SC65, and GR-ON81A were assessed based on their electron-donating ability to 1,1-diphenyl-2-picrylidrazil (DPPH), (Figure 1). GR-SC65 (90.5 ± 2.1%) and GR-ON81A (86.4 ± 4.0%) showed a higher DPPH radical scavenging activity than GR (57.4 ± 1.8%), (*p* < 0.001 vs. GR), which is consistent with the TPC result.

### 2.2. PM Decreases the Viability of A549 Cells

**Figure 2:**
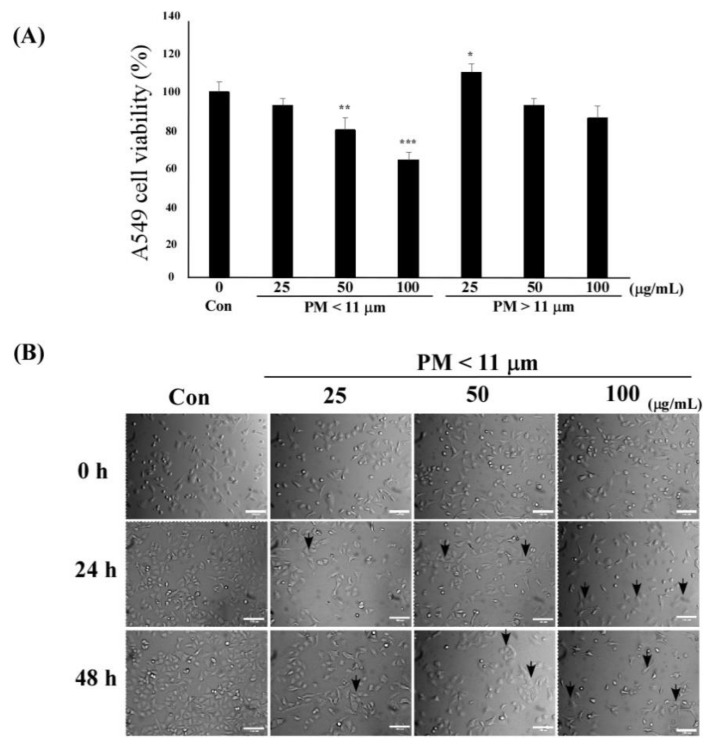
Effects of particulate matter (PM) on the viability and morphology of A549 cells. (**A**) A549 cells were exposed to control or 25, 50, and 100 μg/mL of PM in dimethyl sulfoxide (DMSO). The viability of A549 cells exposed to 25, 50, and 100 μg/mL PM (<11 μm) was determined by the cell counting kit-8 (CCK-8) assay. Values are expressed as mean value ± standard deviation (SD), based on triplicate samples from three independent experiments. Statistically significant differences between groups were analyzed using one-way ANOVA/Dunnett’s *t*-test (* *p* < 0.05, ** *p* < 0.01, and *** *p* < 0.001 vs. Control). (**B**) PM-exposed A549 cells were observed under the microscope and charge-coupled device (CCD) camera after exposure to PM (<11 μm). Arrows indicate morphological changes. Morphology of A549 cells exposed to PM was examined using Nikon Eclipse Ti microscopy (Nikon Instruments Incorporated, Melville, NY, USA) and a CCD camera (Point Grey Research Inc., Richmond, BC, Canada) (Magnification 100×, Scale bar 100 μm).

To examine the effect of PM on type II lung alveolar epithelial cell (A549) viability, we performed the cell counting kit-8 (CCK-8) assay after a 24-h exposure to different sizes (<11 μm and >11 μm) and concentrations (25, 50, 100 μm) of PM. PM easily penetrates the respiratory tract and precipitates in the lung alveoli, which may result in lung damage and dysfunction. PM (<11 μm, 50–100 μg/mL) decreased A549 cell viability when compared with the untreated control (** *p* < 0.01, *** *p* < 0.001). The viability of PM (<11 μm)-treated A549 cells was significantly lower than that observed with PM > 11 μm in size (** *p* < 0.01, *** *p* < 0.001) (Figure 2A). Therefore, PM < 11 μm in size was used for further experiments.

Furthermore, we observed morphological changes in A549 cells after exposure to different concentrations of the PM extract for 0−48 h (Figure 2B). The A549 cells were irregular, shrunken, and detached from the bottom of the cell culture plate after treatment with 25−100 μg/mL PM.

### 2.3. GR-SC65 Suppresses PM-Induced A549 Cell Death

**Figure 3:**
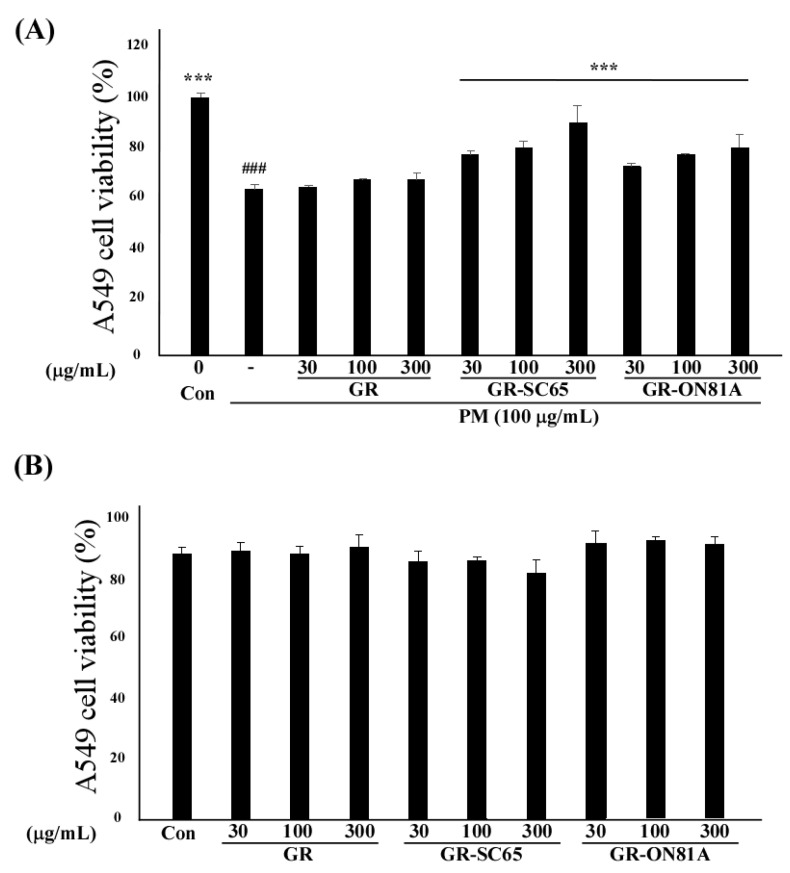
Effect of GR-SC65 on A549 cell viability exposed to particulate matter (PM). (**A**) Effect of GR, GR-SC65, and GR-ON81A on the viability of cultured A549 cells exposed to PM (100 μg/mL). Based on the CCK-8 assay results, 100 μg/mL PM decreases the viability of A549 cells when compared with the untreated cells, but pretreatment with 100 μg/mL GR-SC65 for 1 h increases the viability of the A549 cells exposed to PM (100 μg/mL). Data are expressed as the mean value ± standard deviation (SD). All values are means obtained three independent experiments. Statistically significant differences between groups were analyzed using one-way ANOVA/Dunnett’s *t*-test (^###^ *p* < 0.001 vs. untreated-A549 cells), (*** *p* < 0.001 vs. PM-treated A549 cells). Statistically significant differences between two groups were analyzed using the two-sample *t*-test. (**B**) Viability of the cultured A549 cells exposed to 30–300 μg/mL GR, GR-SC65, and GR-ON18A was determined by the CCK-8 assay. The CCK-8 assay results revealed that incubation for 48 h with 300 μg/mL GR and 300 μg/mL GR-ON18A decreases the viability of A549 cells. Data are expressed as the mean ± standard deviation (SD). All values are means obtained from three independent experiments. Statistically significant differences between groups were analyzed using one-way ANOVA/Dunnett’s *t*-test (^###^ *p* < 0.001 vs. untreated-A549 cells).

Next, we investigated whether GR fermented with different LAB can suppress PM-induced A549 cell death. GR-LAB extracts (GR-SC65 and GR-ON81A) significantly increased PM-treated A549 cell viability in a dose-dependent manner between 30–300 μg/mL (*** *p* < 0.001 vs. PM-treated A549 cells; Figure 3A). GR-SC65 significantly decreased PM-induced A549 cell death when compared with PM (*** *p* < 0.001 vs. PM). In addition, 100 μg/mL GR-SC65 significantly inhibited PM-induced A549 cell death, when compared with 100 μg/mL of GR (^$$$^ *p* < 0.001 vs. GR). Therefore, 100 μg/mL GR-SC65 was employed in further experiments. No cytotoxic effect of GR, GR-SC65, or GR-ON81A was observed on the A549 cell viability after treatment with 30–300 μg/mL of the extract (^###^ *p* < 0.001 vs. control) (Figure 3B).

### 2.4. GR-SC65 Reduces PM-Induced ROS Generation in A549 Cells

**Figure 4:**
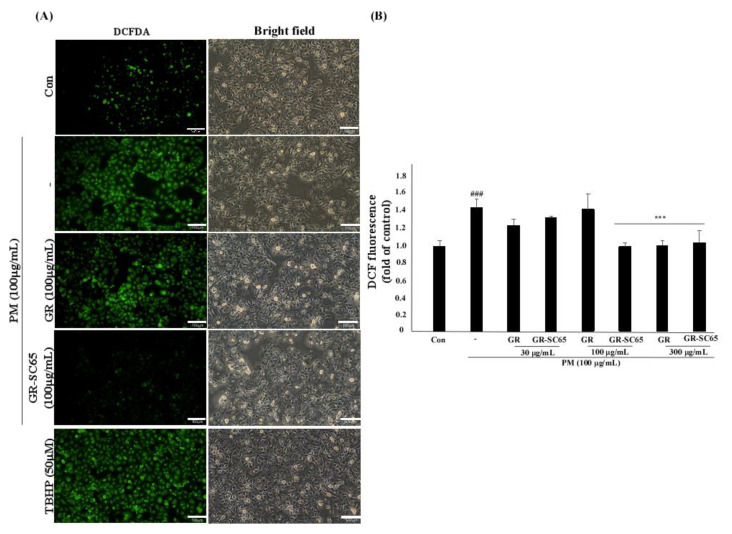
Scavenging effect of GR-SC65 on PM-induced intracellular ROS in A549 cells. (**A**) Intracellular ROS were detected by fluorescence microscopy (Nikon Eclipse Ti microscope, Point Grey Research, Richmond, BC, Canada) after 2-7-dichlorodihydrofluorescein diacetate (DCF-DA) staining. Tert-Butyl hydroperoxide (TBHP), a ROS inducer, was used as a positive control. (**B**) ROS scavenging effect of 30−300 μg/mL of GR and GR-SC65 on PM-induced ROS in A549 cells. The ROS degeneration ability was measured using DCF-DA, as described in the Methods section, at a wavelength of 485/535 (Ex/Em). Statistically significant differences between groups were analyzed using one-way ANOVA/Dunnett’s *t*-test (^###^ *p* < 0.001 vs. untreated-A549 cells and *** *p* < 0.001 vs. PM). PM, particulate matter; ROS, reactive oxygen species; DCF-DA, 2,7-dichloro-dihydro-fluorescein diacetate.

PM generates ROS, which activate ROS-mediated cellular signaling that leads to cell death and inflammation [26]. To determine the ROS radical scavenging activity of GR-SC65 against PM-induced ROS, we measured the intracellular ROS level using 2-7-dichlorodihydrofluorescein diacetate (DCF-DA). The increased fluorescence intensity indicated that PM significantly induced a 1.5 ± 0.1-fold increase in intracellular ROS generation when compared with the control (^###^ *p* <0.001 vs. control). As shown in Figure 4, data indicated that GR-SC65 significantly induced a 0.7 ± 0.2-fold decrease in intracellular ROS generation when compared with PM (*** *p* <0.001 vs. PM). Furthermore, GR-SC65 significantly induced a 0.7 ± 0.0-fold decrease in intracellular ROS generation when compared with GR (*** *p* <0.001 vs. GR). 

### 2.5. GR-SC65 Protects A549 Cells from PM-Induced Apoptotic Cell Death

**Figure 5:**
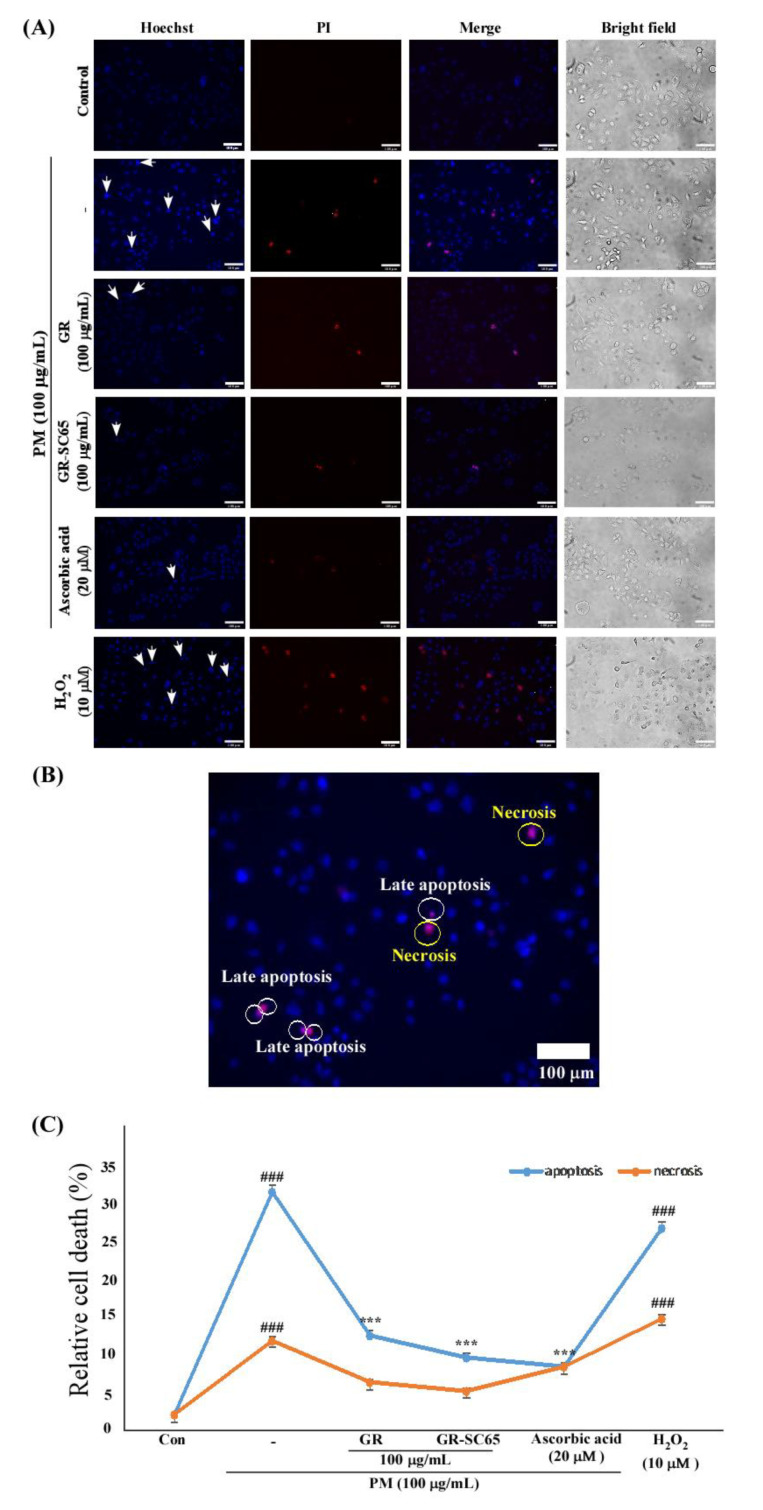
GR-SC65 attenuates the particulate matter (PM)-induced apoptotic cell death in A549 cells. (**A**) A549 cells were treated with each sample for 24 h. Apoptotic cells were detected by Hoechst 33342 and propidium iodide (PI) nuclear staining. Apoptotic cells stained by Hoechst 33342 and PI were detected by confocal microscopy with Metamorph software (Universal Imaging, West Chester, PA, USA; Magnification = 200×, Scale bar = 100 μm). White arrows indicate the apoptotic, shrunken, and hyperchromic cells. Statistically significant differences between groups were analyzed using one-way ANOVA/Dunnett’s *t*-test (^###^ *p* < 0.001 vs. untreated-A549 cells), (*** *p* < 0.001 vs. PM-treated A549 cells). (**B**) The purple merged images with both Hoechst 33342 and PI dye indicate late apoptotic cells or necrotic cells. (**C**) Relative apoptotic or necrotic cells were calculated as a percentage of the total number of cells. Relative apoptotic cells (%) = (the number of Hoechst 33342 positive with chromatin condensation and PI-positive cells/total cells counted with Hoechst 33342 staining) × 100. Relative necrotic cells (%) = (the number of Hoechst 33342 positive with nucleus swelling and PI-positive cells/total cells counted with Hoechst 33342 staining) × 100. Data are expressed as the mean value ± standard deviation (SD). All values are means obtained from three independent experiments. Statistically significant differences between groups were analyzed using one-way ANOVA/Dunnett’s *t*-test (^###^ *p* < 0.001 vs. control), (*** *p* < 0.001 vs. PM).

To determine whether GR-SC65 could block PM-induced apoptotic or necrotic cell death, A549 cells were stained with Hoechst 33342 and propidium iodide (PI) to detect apoptosis or necrosis. Hoechst 33342 is a cell-permeable dye that stains the nuclei of both live and dead cells by intercalating in the minor groove of DNA. PI is a membrane-impermeable dye that stains dead cells by binding in between DNA bases. Typical apoptotic features are chromatin condensation and nuclear cleavage, while morphological features of necrosis include cellular and organelle swelling, as well as cell membrane disruption. PI stains late apoptotic and necrotic cells, but not early apoptotic cells. We observed an increased Hoechst 33342 fluorescence intensity in PM-treated A549 cells owing to chromatin condensation when compared with the control. In PM- and H_2_O_2_-treated A549 cells, early apoptotic cells were observed, which are indicated with white arrows in images of Hoechst staining. In contrast, treatment with GR-SC65 and ascorbic acid decreased the number of early apoptotic cells induced by PM (Figure 5A). The purple merged images with both Hoechst 33342 and PI dye indicate late apoptotic cells or necrotic cells. Late apoptotic cells were stained purple following nuclear cleavage, and necrotic cells were stained purple with cellular and organelle swelling. The population of apoptotic cells was increased by PM treatment (30.1 ± 0.9%), when compared with the control (^###^ *p* < 0.001 vs. control). Apoptotic cells were significantly decreased by 7.6 ± 0.6% following GR-SC65 treatment when compared with PM (*** *p* < 0.001 vs. PM); however, necrotic cells were not significantly reduced (*** *p* < 0.001 vs. PM), (Figure 5B). These findings suggested that GR-SC65 reduces PM-induced apoptotic cell death.

### 2.6. GR-SC65 Inhibits PM-Induced ROS-Dependent Apoptosis by Downregulating Active Caspase Protein Expression

**Figure 6:**
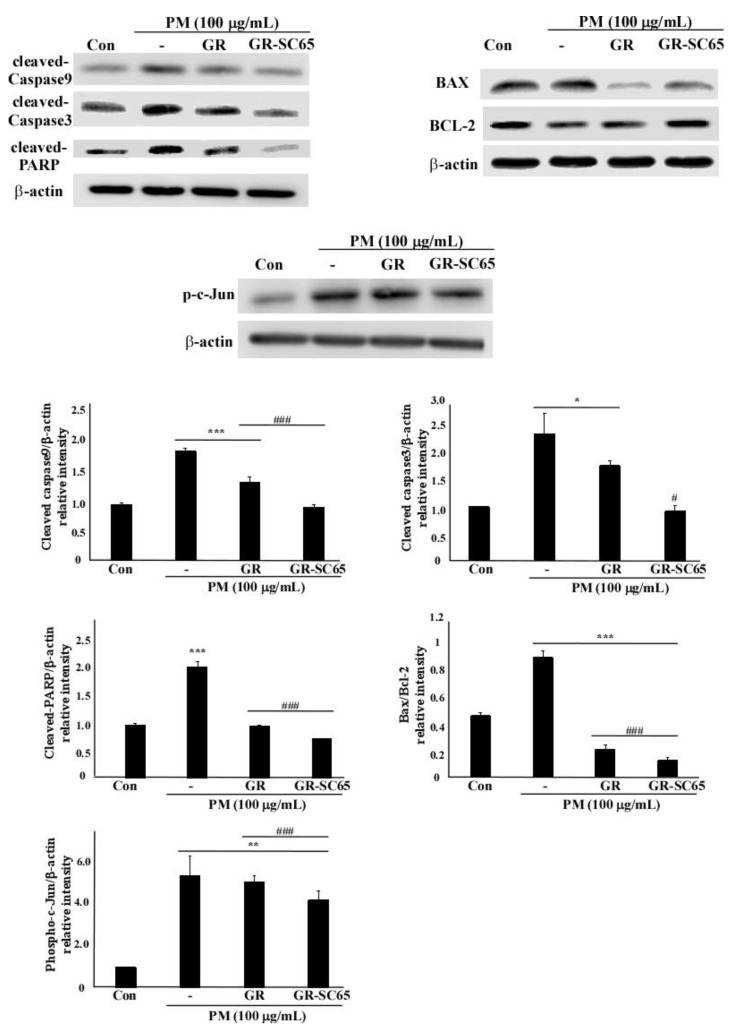
GR-SC65 inhibits particulate matter (PM)-induced A549 cell apoptosis through apoptotic pathways. A549 cells were pre-treated with 100 μg/mL GR or GR-SC65 for 1 h, followed by treatment with 100 μg/mL PM for 24 h. Whole-cell lysates were processed for western blot analysis and probed with indicated antibodies. Active caspase-3, active caspase-9, active PARP, BAX, and BCL-2 protein expression levels in A549 cells were detected using western blotting. Relative intensity is shown as the means ± standard deviation (SD) of three independent experiments. ^#^ *p* < 0.05 and ^###^ *p* < 0.001 using ANOVA test for the comparison between control and PM-treated A549 cells. *** *p* < 0.001, ** *p* < 0.01, and * *p* < 0.05 using ANOVA test for the comparison between PM-treated A549 cells and other GR or GR-SC65 treated A549 cells. PARP, poly (ADP-ribose) polymerase.

To elucidate the anti-apoptotic mechanism of GR-SC65, we determined the levels of active caspase-9 and -3, poly (ADP-ribose) polymerase (PARP), BCL-2, and BAX protein expression (Figure 6). The levels of active caspase-9, caspase-3, and PARP proteins were decreased after GR-SC65 treatment of PM-induced A549 cells (^#^ *p* < 0.05, ^###^ *p* < 0.001 vs. PM). As shown in Figure 6, the levels of the anti-apoptotic protein BCL-2 increased by 28.2 ± 5.5% after GR-SC65 treatment when compared with PM. In contrast, the levels of the apoptotic protein BAX were decreased by 83.0 ± 0.0% after GR-SC65 treatment when compared with PM. Furthermore, the ratio of BAX and BCL-2 protein levels was decreased by 86.7 ± 4.1% after GR-SC65 treatment when compared with PM (^###^ *p* < 0.001 vs. PM). The level of phospho-c-Jun protein was significantly decreased by 22.7 ± 5.5% after GR-SC65 treatment (^###^ *p* < 0.001 vs. PM). Collectively, these data suggest that GR-SC65 reduced apoptotic cell death in PM-exposed A549 cells.

### 2.7. Comparison of Peptide Sequence of GR and GR-SC65 

**Figure 7:**
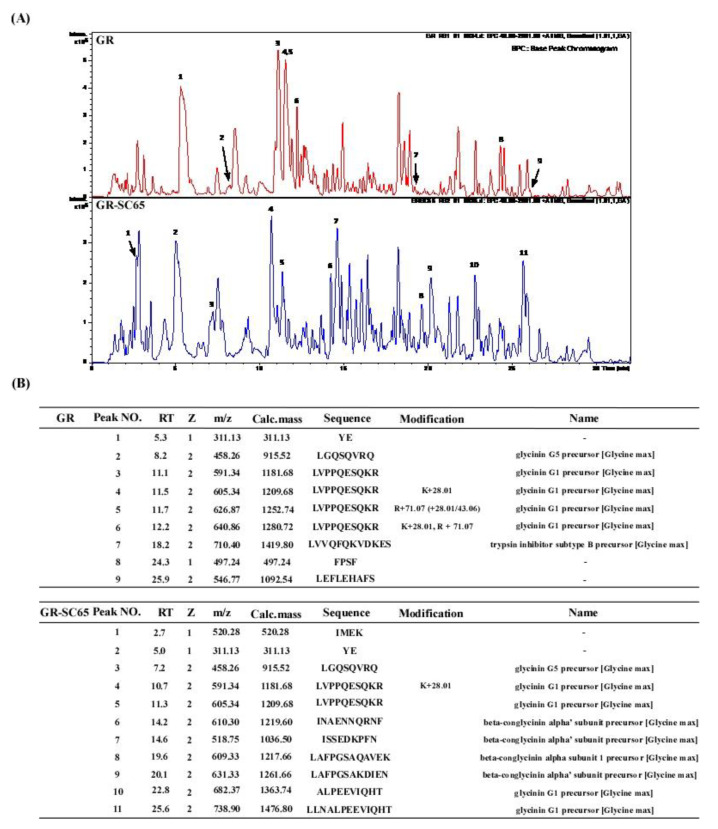
Soy peptide qualification in GR and GR-SC65 by LC/MS analysis (**A**) LC/MS chromatogram of GR and GR-SC65. Peak numbers refer to (**B**). (**B**) Peptides quantified by in GR and GR-SC65 by LC/MS. LC/MS, liquid chromatography-mass spectrometry.

The major proteins in soybean are β-conglycinin and glycinin (>85%). Glycinin and α- and α′-subunits of β-conglycinin are preferred substrates for several LAB strains [27]. We detected four peptides (INAENNQRNF, ISSEDKPFN, LAFPGSAQAVEK, and LAFPGSAKDIEN) in GR-SC65, but not in GR, using liquid chromatography-mass spectrometry (LC/MS) analysis (Figure 7). These four peptides were identified using the BLAST database, which included β-conglycinin breakdowns [28]. Furthermore, these four soy peptides in GR-SC65 possess rich aromatic (H, F, W, Y) and hydrophobic (V, I, and L) amino acid residues, typically observed in antioxidant peptides [29]. This result suggested that the enhanced antioxidant effect of GR-SC65 in PM-induced A549 cells may be attributed to the increased presence of antioxidant peptides when compared with GR. 

## 3. Discussion

PM contributes to the development of various diseases, including heart disease, stroke, chronic obstructive pulmonary disease, lung cancer, and acute respiratory infections [30]. Reportedly, over two million deaths are known to occur worldwide annually owing to asthma, chronic obstructive pulmonary disease, pulmonary fibrosis, and acute lung injury after severe PM exposure [31]. In particular, PM less than 10 μm can easily penetrate the respiratory tract and deposit in the lower respiratory tract, leading to pulmonary structural and functional damage [32].

Previously, *R. nulubilis* (RN) cultivated with *Ganoderma lucidum* mycelium has shown increased antioxidant and anti-inflammatory activity when compared with unfermented RN [20]. Consistent with these studies, we observed that the DPPH scavenging activity, TPC, antioxidant peptides, and aglycone form of isoflavones (daidzein, glycitein, and genistein) were significantly increased in GR after fermentation with LAB [33]. The increased antioxidant activity of GR-SC65 could be attributed to the increased content of isoflavone aglycones (daidzein, glycitein, and genistein) [34] and four soy β-conglycinin peptides (INAENNQRNF, ISSEDKPFN, LAFPGSAQAVEK, and LAFPGSAKDIEN) [35,36].

Alveolar epithelial cells consist of type I alveolar epithelial cells (AT1) and type II alveolar epithelial cells (AT2). AT2 are progenitor cells for both AT1 and AT2 and can differentiate into AT1, which is important for pulmonary epithelial tissue recovery after injury. Continuous damage to alveolar epithelial cells can contribute to the development of pulmonary fibrosis, asthma, and chronic obstructive pulmonary disease [37]. In the present study, we investigated whether prophylactic treatment of GR fermented with Lactobacillus could reduce type II alveolar epithelial (A549) cell death induced by PM. Herein, we aimed to investigate the protective actions of GR-SC65 against PM-induced type II alveolar epithelial (A549) cell death.

Recent studies have demonstrated that exposure to ambient airborne PM, with an aerodynamic diameter less than 10 μm (PM10), causes cell death in A549 cells and keratinocytes [38,39,40]. In A549 cells, the water-insoluble fraction of PM was reportedly more toxic than the equivalent dose of PM [41]. In the present study, PM was extracted with 75% ethanol (EtOH). Our results revealed that the 75% ethanol extract of PM was more toxic than the water extract of PM in A549 cells. PM-induced cell death was reduced after GR-SC65 treatment when compared with that observed after PM and GR-ON81A treatment (*p* < 0.001 vs. PM). We postulated that GR-SC65 would exhibit anti-cell death activity; therefore, we investigated its effect on PM-induced ROS and cell death.

Several studies have demonstrated that long-term PM exposure (>100 μg/mL) induces oxidative stress, resulting in lung toxicity, perturbation of the mitochondrial permeability transition pore, and disruption of the electron transport chain, thus triggering cellular apoptosis or necrosis in the lungs, and eventually leading to acute lung injury and chronic obstructive pulmonary disease [42,43,44]. According to the report by Samara and Jiang, water-soluble PMs contain ionic species (NO^3−^, SO_4_^2−^, Cl^−^, Na^+^, NH_4_^+^, K^+^, Mg^2+^, Ca^2+^), water-soluble organic carbon (WSOC), organic compounds (polycyclic aromatic hydrocarbons (PAHs)), and trace elements (Al, As, Ba, Cd, Cr, Cu, Fe, Pb, Mn, Ni, Zn, Pt, Pd, Rh, Ru, Ir, Ca, and Mg), and water-insoluble PMs contain metal oxide nanoparticles, kaolinite, calcium carbonate, and some organic carbon [45,46]. It is presumed that both water-soluble (ZnO, CuO, Fe, PAHs) and water-insoluble (SiO_2_, TiO_2_) components could be present in our 75% EtOH PM extracts [47]. For instance, PAHs in PM activate the membrane-bound NADPH oxidase complex in the plasma membrane and generate superoxide anions (O_2_^−^) [48,49]. In addition, PAHs (AHR ligands) in PM activate the AHR, inducing AHR to translocate to the nucleus and bind to DNA elements (XRE), inducing CYP enzymes [50]. For example, CYP1 expression contributes to ROS formation, including O_2_^−^ through PAHs and other organic chemicals. Goto et al. have reported that genistein represses ROS generation and NADPH oxidase (NOX) activity, suggesting that it downregulates ROS such as H_2_O_2_ and O_2_^−^ [51]. Our previous studies have reported that the aglycone form of isoflavones (daidzein, glycitein, and genistein) increases in GR after fermentation with *L. pentosus* SC65, which reportedly possesses anti-inflammatory and antioxidant effects [10,33,52]. These studies imply that GR-SC65 might downregulate PM-induced ROS, including H_2_O_2_ and O_2_^−^, by suppressing NOX activity. Transition metals such as Cu^+^ and Fe^2+^ in PM are responsible for producing hydroxyl radicals (OH^−^) in the extracellular fluid through redox cycling (Fenton or Haber-Weiss reactions) [53]. In particular, reactive OH^−^ is the most harmful radical among ROS, leading to lipid peroxidation, excessive oxidative stress, and oxidative DNA damage [54]. Aglycone forms of iso-flavonoids (daidzein, genistein, and glycitein) act as powerful chelating agents for iron (II) metal cations through the direct transfer of a hydrogen atom to prevent the generation of hydroxyl radicals in the extracellular fluid [55]. We observed that GR-SC65 significantly reduced PM-induced intracellular ROS levels in A549 cells. It can be postulated that PM compounds such as ZnO, CuO, Fe, PAHs, SiO_2_, and TiO_2_ produce ROS (·OH, RO_2_·, O_2_^−^, and H_2_O_2_), which was reduced by GR-SC65 treatment. We observed that the antioxidant physiological activities of GR-SC65 were increased after fermentation with LAB, and GR-SC65 decreased the level of PM-induced intracellular ROS expression. In agreement with these results, the aglycone form of isoflavones (daidzein, glycitein, and genistein) in GR-SC65 might have a suppressive effect on the level of intracellular ROS in A549 cells. ROS overproduction damages nucleic acids, lipids, proteins, membranes, and organelles, leading to cell death such as apoptosis [56]. These findings prompted us to investigate whether GR-SC65 can suppress ROS-induced cell death associated with apoptosis.

**Figure 8:**
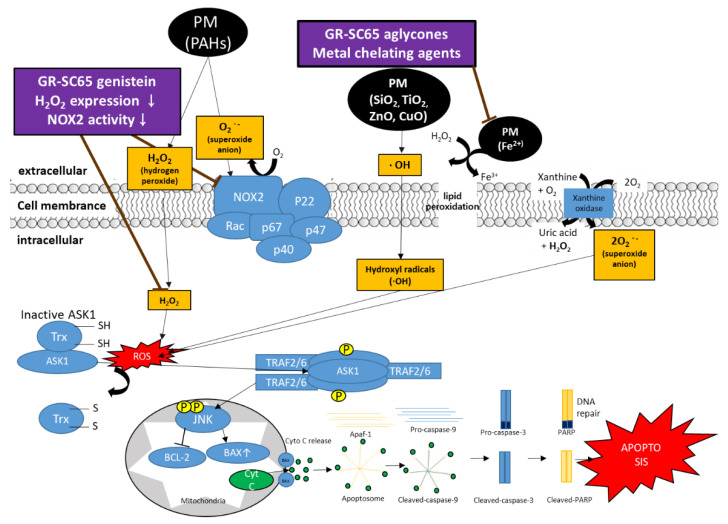
GR-SC65 suppresses the PM-induced ROS-dependent apoptosis pathway. GR-SC65 suppresses PM-induced ROS (hydrogen peroxide, superoxide anions, and hydroxyl radicals). The levels of BCL-2 proteins are upregulated by GR-SC65. GR-SC65 also inhibits the activation of BAX, caspase-9, caspase-3, and PARP, all downstream signaling molecules. The brown arrow indicates the inhibitory activity of GR-SC65 through the PM-induced ROS-mediated apoptosis pathway. PM, particulate matter; ROS, reactive oxygen species; PAHs, polycyclic aromatic hydrocarbons; PARP, poly (ADP-ribose) polymerase; NOX2, NADPH oxidase 2.

In the present study, our results confirmed that GR-SC65 significantly inhibited apoptotic cell death rather than necrotic cell death (Figure 5) [57]. Therefore, we investigated whether GR-SC65 reduces signaling molecules related to PM-induced apoptotic cell death. ROS (H_2_O_2_) activates apoptosis signal-regulated kinase 1 (Ask1) by oxidizing disulfide reductase thioredoxin-1 (Trx1). Following Trx1 oxidation, it dissociates from Ask1, which induces auto-phosphorylation, and sequentially activates Jun-N-terminal kinase (JNK) [58]. Activated JNK accumulates active BAX in the outer mitochondrial membrane. BAX triggers apoptosis by forming pores within the mitochondrial outer membrane, resulting in the release of cytochrome c (cyt c) into the cytosol [59]. Following the release into the cytosol, cyt c triggers the activation cascade of caspases. The level of the anti-apoptotic BCL-2 protein in GR-SC65 was increased to a greater extent than that in the PM and GR; conversely, the level of the apoptotic BAX protein in GR-SC65 was downregulated. Cyt c binds to apoptotic protease-activating factor-1 (Apaf-1) to form the apoptosome, which recruits and activates caspase-9. Activated procaspase-9 produces effector caspases-3 that cleaves PARP, a hallmark of apoptosis [60]. In concert with decreased PM-induced apoptotic cell death in GR-SC65 treated A549 cells, we observed decreased levels of active caspase-9, caspase-3, and PARP proteins in GR-SC65 treated A549 cells. It is plausible that the aglycone form of isoflavones (daidzein, glycitein, genistein) and the four soy β-conglycinin peptides (INAENNQRNF, ISSEDKPFN, LAFPGSAQAVEK, and LAFPGSAKDIEN) present in GR-SC65 suppress excessive ROS production or scavenge ROS, suppressing PM-induced apoptotic signaling molecules. In summary, GR-SC65 scavenged PM-induced excessive ROS expression and attenuated PM-induced apoptosis; therefore, it might act as a ROS scavenger against oxidative stress (Figure 8). Future studies should assess whether GR-SC65 is effective in the ROS-independent apoptosis mechanism.

## 4. Materials and Methods

### 4.1. Preparation of the Extract of Germinated Rhynchosia nulubilis (GR) Fermented with LAB

GR fermented with LAB was provided by Dr. Park from the Cell Activation Research Institute (CARI Co., Ltd., Seoul, Republic of Korea). *Lactobacillus pentosus* SC65 and *Pediococcus pentosaceus* ON81A were isolated from pickled burdock and onion, respectively [33]. GR (20% *w/v*) was extracted with distilled water at 121 °C for 15 min and then inoculated with *L. pentosus* SC65 and *P. pentosaceus* ON81A strains (0.02% *w/v*). Then, 20% of the liquid phase of the fermentation product was mixed with GR broth. The mixture was fermented at 30 °C for 24 h. GR-inoculated probiotic strain SC65 (GR-SC65) and ON81A (GR-ON81A) were inactivated for 12 h using a freeze dryer (Ilshin Co., Ltd., Dongducheon-si, Gyeonggi-do, Republic of Korea). Solid-phase fermented GR-SC65 and GR-ON81A were extracted for 24 h at 50 °C with 70% ethanol (10% *w/v*). 

### 4.2. PM Collection and Extraction

**Figure 9:**
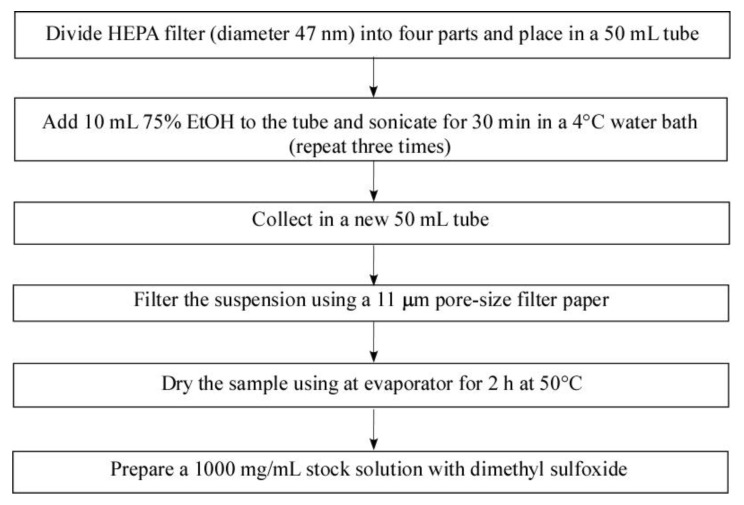
Schematic diagram of PM extraction. PM sampling was performed at the Functional Food Laboratory at Gachon University for 12 months (June 2016–June 2017; Seoungnam si, Gyeonggi-do, Republic of Korea). PM samples were filtered using an 11 μm pore-size filter paper (1001−110, Whatman, Maidstone, Kent, England).

PM sampling was performed at the Functional Food Laboratory at Gachon University for 12 months (June 2016–June 2017; Seoungnam si, Gyeonggi-do, Republic of Korea). To obtain PM particles for in vitro experiments, HEPA filters were soaked with 10 mL of 75% alcohol in a 50 mL tube, followed by sonication for 30 min using a 4 °C water ultrasonic bath sonicator (Powersonic 610, KLEENTEK, Maroochydore, Queensland, Australia). This procedure was repeated three times. The suspension layer was collected in a new 50 mL tube and then <11 sized PM were filtered using filter papers (1001−110, Whatman, Maidstone, Kent, England). The filtered PM suspension was concentrated for 48 h using a freeze-drier, resulting in a yield (*w/w*) of 7.85% (Figure 9), [61].

### 4.3. Determination of Total Polyphenol Contents

The total polyphenol content (TPC) of GR, GR-SC65, and GR-ON81A was determined using a previously described method [33]. The reaction mixture was composed of 50 μL of sample extracts, 100 μL of 1N Folin-Ciocalteu reagent, and 75 μL of 7% sodium carbonate solution. Gallic acid was used as the standard. The absorbance was measured at 720 nm after a 30 min reaction in a dark chamber at room temperature. The results were reported as milligram gallic acid equivalent per gram (mg GAE/g) of GR, GR-SC65, and GR-ON81A.

### 4.4. DPPH Free Radical Scavenging Activity

The DPPH assay was performed according to a previously detailed protocol [33]. In brief, 100 μL of a 0.2 mM 1,1-diphenylpicrylhydrazyl (DPPH) methanol solution was added to 100 μL of GR, GR-SC65, GR-ON81A, and ascorbic acid (350 μM) solutions, respectively, and then allowed to react at room temperature. After 30 min of incubation, the optical density was measured at 517 nm and converted into radical scavenging activity (%) using the following formula:

Radical scavenging activity (%) = (Absorbance of the control − Absorbance of the test sample) × 100

### 4.5. Cell Culture

A549 cells were obtained from ATCC (Manassas, VA, USA). The cells were cultured at 37 °C in a humidified atmosphere containing 5% CO_2_. A549 cells were maintained in 75 cm^2^ culture flasks with RPMI 1640 (Welgene, Seoul, Republic of Korea), supplemented with 10% fetal bovine serum (FBS; Welgene, Seoul, Republic of Korea) and 100 U/mL penicillin and streptomycin (Welgene, Seoul, Republic of Korea).

### 4.6. Morphology

Cells were viewed and counted using an eclipse Ti-S inverted microscope (Nikon, Tokyo, Japan) at 100× magnification. Images were obtained using the Metamorph software (Universal Imaging, West Chester, PA, USA). The images presented are representative of three independent experiments.

### 4.7. Cell Viability

A549 cells were evaluated using the cell counting kit-8 (CCK-8) assay (Dojindo Laboratories, Kumamoto, Japan), as described previously [62]. In brief, cells (1 × 10^4^ cells/well) were plated onto a 96 well plate and treated with various concentrations (30, 100, and 300 μg/mL) of GR, GR-SC65, and GR-ON81A for 48 h. The CCK-8 solution was added, and the cells were incubated for 2 h. After adding the CCK-8 solution, the percentage cell viability was measured using a microplate spectrophotometer (Epoch, BioTek Instruments, Inc., Winooski, VT, USA) at 450 nm.

### 4.8. Reactive Oxygen Species (ROS) Assay

Levels of intracellular ROS were determined using 2,7-dichloro-dihydro-fluorescein diacetate (DCFH-DA), which reacts with ROS to form the fluorescent product 2,7-dichlorodihydrofluorescein (DCF), in accordance with the manufacturer’s protocol (ab113851, Abcam, Cambridge, United Kingdom). In brief, A549 cells (1 × 10^4^ cells) were incubated for 18 h, washed once with 1x dilution buffer, and incubated with 25 μM DCFH-DA for 45 min. Cells were treated with GR and GR-SC65 for 2 h after washing with 1× dilution buffer. Then, the cells were treated with PM (100 μg/mL) for 3 h. Images of DCF-DA fluorescence were captured using a fluorescence microscope (Nikon Eclipse Ti microscope, Point Grey Research, Richmond, BC, Canada). The fluorescence intensity of DCFH-DA was measured using a fluorescence plate reader (EnSpire, PerkinElmer, Waltham, MA, USA) at an excitation wavelength of 485 nm and an emission wavelength of 535 nm. DCF fluorescence values were normalized to the total cell viability detected by the CCK-8 assay. Tert-butyl hydroperoxide (TBHP) was used as a positive control to induce ROS production. The data are expressed as a fold of the control.

### 4.9. Hoechst 33342 and PI Double Staining

In brief, the cells were plated in 12-well plates and incubated for 24 h. Experiments were performed in five groups: control, PM, GR + PM, GR-SC65 + PM, and H_2_O_2_. A549 cells were pre-treated with GR or GR-SC65 (100 μg/mL) for 1 h and then stimulated with PM (100 μg/mL).

The cells were washed with PBS three times and stained with Hoechst 33342 (1 mg/L) for staining cellular DNA and propidium iodide (PI, 1 mg/L) for staining of damaged cells bound to DNA. Images of Hoechst 33342 and propidium iodide fluorescence were captured using fluorescence microscopy after the cells were washed with PBS three times. The fluorescence staining percentage of positive cells was calculated based on the obtained images.

### 4.10. Western Blot Assay

Western blotting was performed as previously described [63]. Briefly, A549 cells (1 × 10^6^) were incubated for 6 h in 6 well plates, pre-treated with GR and GR-SC65 (100 μg/mL) for 1 h, followed by treatment with PM (100 μg/mL). After incubation for 24 h, the cells were lysed using radioimmunoprecipitation assay (RIPA) buffer (Cell Signaling, Danvers, MA, USA) and sonicated. The samples were centrifuged at 14,000× *g* for 10 min to pellet the cell debris; then, the supernatant was transferred to a fresh microfuge tube. Protein concentrations were determined using the Pierce bicinchoninic acid (BCA) protein assay kit (Thermo Fisher Scientific, Waltham, MA, USA). Equal amounts of protein were separated by 10% sodium dodecyl sulfate-polyacrylamide gel electrophoresis (SDS-PAGE). Proteins were transferred to nitrocellulose membranes (Bio-Rad Laboratories, Inc., Hercules, CA, USA) and blocked with 5% non-fat milk for 1 h at room temperature, followed by overnight incubation at 4 °C with Tris-buffered saline containing Tween (TBS-T, 20 mM Tris, 500 mM sodium chloride [pH 7.6], and 0.1% Tween 20), and 5% bovine serum albumin, anti-cleaved caspase 9 (1:1000; Cell Signaling, Danvers, MA, USA), anti-cleaved caspase 3 (1:1000; Cell Signaling, Danvers, MA, USA), anti-PARP (1:1000; Cell Signaling, Danvers, MA, USA), anti-BAX (1:1000; Cell Signaling, Danvers, MA, USA), anti-BCL-2 (1:1000; Cell Signaling, Danvers, MA, USA), and anti-phosphorylated(p)-c-Jun (1:1000; Cell Signaling, Danvers, MA, USA). The membranes were washed three times for 10 min with TBS-T and then incubated for 1 h with horseradish peroxidase (HRP)-linked anti-rabbit IgG (1:2000; Cell Signaling, Danvers, MA, USA) or anti-mouse IgG (1:2000; Cell Signaling, Danvers, MA, USA). The blots were detected using an enhanced chemiluminescence western blotting detection system with the Odyssey LCI Image software (LI-COR Biosciences, Lincoln, NE, USA). The blots presented are representative of at least three repeats.

### 4.11. Peptide Analysis by LC-MS

The mobile phase for chromatographic separation consisted of solvent A (0.2% formic acid in water, *v/v*) and solvent B (0.2% formic acid in acetonitrile, *v/v*), performed in a gradient manner. Linear gradient elution was performed from 5% B to 95% B in 40 min at a flow rate of 0.2 mL/min. The column compartment was maintained at 30 °C and the injection volume was 5 μL. The analysis was performed using a MicroQ-TOF III mass spectrometer (Bruker Daltonics Inc., Billerica, MA, USA). Additionally, the eluent was monitored by electrospray ion mass spectrometry (ESI-MS) in positive ion mode, and the sample was scanned from *m*/*z* 50 to 2000. A homologous peptide search was performed using the *Glycine max* genome sequence database from a protein BLAST search of the National Center for Biotechnology Information (NCBI) database (www.ncbi.nlm.nih.gov/blast, accessed on 6 April 2020).

### 4.12. Statistical Analysis

Data were obtained from at least three independent experiments and are expressed as mean value ± standard deviation (SD). Statistical analyses were performed using one-way analysis of variance (ANOVA) with Dunnett’s post-hoc test. A two-sample *t*-test was used for comparing two groups. Data were analyzed using the SPSS v12 software (IBM Corp., Chicago, IL, USA).

## 5. Conclusions

We demonstrated that GR fermented with *L. pentosus* SC65 (GR-SC65) has a significant antioxidant and anti-apoptotic effect against PM-induced intracellular ROS and apoptotic cell death. GR-SC65 showed the highest DPPH activity and TPC among GR, GR-SC65, and GR-ON81A. GR-SC65 reduced overexpressed intracellular ROS level and increased A549 cell viability against PM treatment. GR-SC65 significantly decreased PM-induced apoptotic death of A549 cells. GR-SC65 reduced the level of apoptosis signaling protein expression (BAX, activated-caspase-9, activated-caspase-3, activated-PARP) and increased the levels of an anti-apoptotic protein (BCL-2), when compared with that induced by GR. Aglycone forms of iso-flavonoid contents (daidzein, glycitein, and genistein) and antioxidant four soy β-conglycinin peptides (INAENNQRNF, ISSEDKPFN, LAFPGSAQAVEK, and LAFPGSAKDIEN) in GR-SC65 scavenged the PM-induced intracellular ROS, resulting in the reduction of the signaling molecules related to ROS-dependent apoptotic cell death. Therefore, we conclude that GR-SC65 can be used as a suitable therapeutic and preventive agent against PM-induced lung injury.

## Figures and Tables

**Table 1:** Changes in total phenolic contents (TPC) of GR, GR-SC65, and GR-ON81A with different extraction temperatures using 70% aqueous ethanol solution.

Temperature [°C]	Sample	Total Phenolic Content (mg Gallic Acid Equivalents (GAE)/g Sample)
27	GR	80.9 ± 0.6
	GR-SC65	113.2 ± 2.3 ***
	GR-ON81A	89.8 ± 0.2
50	GR	93.8 ± 1.3
	GR-SC65	161.6 ± 1.9 ^###^
	GR-ON81A	158.8 ± 2.8 ^###^

GR, unfermented black soybeans; GR-SC65, L. *pentosus* SC65 fermented black soybeans at 37 °C for 24 h; GR-ON81A, P. *pentosaceus* ON81A fermented black soybeans at 37 °C for 24 h. Data are expressed as the mean value of replication (*n*) ± standard deviation (SD). Statistically significant differences between groups were analyzed using one-way ANOVA/Dunnett’s *t*-test (*** *p* < 0.001 vs. GR extracted with 27 °C of 70% EtOH, ^###^ *p* < 0.001 vs. GR extracted with 50 °C of 70% EtOH).

## Data Availability

Data sharing not applicable.
